# The Development of DNA Based Methods for the Reliable and Efficient Identification of* Nicotiana tabacum* in Tobacco and Its Derived Products

**DOI:** 10.1155/2016/4352308

**Published:** 2016-08-18

**Authors:** Sukumar Biswas, Wei Fan, Rong Li, Sifan Li, Wenli Ping, Shujun Li, Alexandra Naumova, Tamara Peelen, Esther Kok, Zheng Yuan, Dabing Zhang, Jianxin Shi

**Affiliations:** ^1^Joint International Research Laboratory of Metabolic & Developmental Sciences, SJTU-University of Adelaide Joint Centre for Agriculture and Health, School of Life Sciences and Biotechnology, 800 Dongchuan Road, Minghan District, Shanghai 200240, China; ^2^Institute of Tobacco Research, Henan Academy of Agricultural Sciences, Xuchang, Henan 461000, China; ^3^Central Customs Laboratory, Bulgarian Customs Agency, G. S. Rakovski Street 47, 1202 Sofia, Bulgaria; ^4^Dutch Customs Laboratory, Kingsfordweg 1, 1043 GN Amsterdam, Netherlands; ^5^RIKILT, Wageningen University and Research Centre, P.O. Box 230, 6700 AE Wageningen, Netherlands

## Abstract

Reliable methods are needed to detect the presence of tobacco components in tobacco products to effectively control smuggling and classify tariff and excise in tobacco industry to control illegal tobacco trade. In this study, two sensitive and specific DNA based methods, one quantitative real-time PCR (qPCR) assay and the other loop-mediated isothermal amplification (LAMP) assay, were developed for the reliable and efficient detection of the presence of tobacco (*Nicotiana tabacum*) in various tobacco samples and commodities. Both assays targeted the same sequence of the uridine 5′-monophosphate synthase (*UMPS*), and their specificities and sensitivities were determined with various plant materials. Both qPCR and LAMP methods were reliable and accurate in the rapid detection of tobacco components in various practical samples, including customs samples, reconstituted tobacco samples, and locally purchased cigarettes, showing high potential for their application in tobacco identification, particularly in the special cases where the morphology or chemical compositions of tobacco have been disrupted. Therefore, combining both methods would facilitate not only the detection of tobacco smuggling control, but also the detection of tariff classification and of excise.

## 1. Introduction 

Tobacco (*Nicotiana tabacum *L.) is cultivated as an important commercial crop worldwide for the production of cigarettes, cigars, and other tobacco products [1]. In 2014, the global tobacco industry sold about 5.6 trillion cigarettes and its market value is around US$744 billion [2]. Because of its massive economic importance, there is increasing illicit tobacco trade including smuggling [3]. Globally, about 657 billion cigarettes valued at around US$40.5 million were smuggled yearly [4]. Illicit tobacco trade is the main hindrance to collecting tobacco excises and taxes, and once eliminated, government revenue would gain at least US$31 billion globally [5].

There are mainly three types of tobacco products on the market: cigarettes, cigars, and fine-cut tobacco (smoking tobacco). A cigarette is a roll of fine-cut tobacco having a wrapper of thin paper. A cigar consists of tobacco, rolled in a binder of tobacco/reconstituted tobacco and an outer wrapper of tobacco. The outer wrapper of a cigar can also be made of reconstituted tobacco, for instance, in cigarillos, a short narrow cigar. For tax and excise purposes, these definitions are used and classification of these tobacco products is therefore mainly based on the composition of the outer wrapper.

Reconstituted tobacco is made from a pulp of mashed tobacco waste [6, 7]. The definition of reconstituted tobacco (Harmonised System, World Customs Organisation) is as follows: “it is made by agglomerating finely divided tobacco from tobacco leaves tobacco refuse or dust, whether or not on a backing (e.g. sheet of cellulose from tobacco stems….” Products with a wrapper of reconstituted tobacco which may consist partly of substances other than tobacco are also seen as cigars/cigarillos. Because the differences of excise duties between cigarettes and cigars are significant in many European countries [8], it is necessary to detect any retail cigars/cigarillos that do not match the definition of a cigar/cigarillo and should therefore be sold as a cigarette. Therefore, a reliable method is needed to detect the presence of tobacco in outer wrappers of these tobacco products.

Tobacco identification is usually based on the detection of the presence of nicotine, neophytadiene [6], vitamin E [9], and microscopic fragments [10]. Although these identification methods are still used, in certain cases (especially in reconstituted tobacco), they often failed to work [7]. In practice, microscopic tobacco fragments may not be detected in reconstituted tobacco, and on the other hand fraudulent actions may include the spraying or impregnation of nicotine and neophytadiene into nontobacco constituents. Thus, alternative methods are required for successful detection and identification of tobacco. In EU customs, officers use trained animals, such as dogs [11–13] and giant African pouched rats (*Cricetomys gambianus*) [13], to successfully detect the smell of tobacco. However, because the odors of tobacco can be readily enhanced or eliminated, this method is likely useful to some extent for tobacco smuggling, but obviously not applicable for tariff classification and for excise, which needs DNA based methods. In a previous study, a quantitative real-time PCR (qPCR) assay based on a putrescine N-methyltransferase (*PMT1*) gene,* PMT1a*, was developed to identify* Nicotiana tabacum* in tobacco products [7]. However, because there is another* PMT1b* gene in tobacco genome and the sequence of* PMT1b *was not available in the database [7], therefore, the primers used for* PMT1a* are likely nonspecific to* PMT1a*. In addition, besides* PMT1*, 3 additional* PMT* (*PMT2–PMT4*) genes with high similarity to each other are present in tobacco genome [7], which makes the use of* PMT1a *as an endogenous reference gene of tobacco less reliable.

In this study, by targeting a putative single copy tobacco endogenous gene sequence of the uridine 5′-monophosphate synthase (*UMPS*), we developed two DNA based methods, a novel quantitative real-time PCR (qPCR) method and a loop-mediated isothermal amplification (LAMP) method, to detect the presence of tobacco components in various commodities. The specificity and sensitivity were assayed using various available materials, including customs samples, reconstituted tobacco samples, cured tobacco samples, and locally purchased cigarettes. Our results showed that the novel qPCR based on* UMPS* had 1.5-fold lower limit of detection and better specificity than the previously reported* PMT1a* based qPCR method [7]. Both qPCR and LAMP developed on* UMPS* could be useful for rapid tobacco identification in a reliable and efficient way.

## 2. Materials and Methods

### 2.1. Plant Materials

All the plant materials used in this study, including different tobacco and nontobacco materials and products, are listed in Table 1.

### 2.2. Genomic DNA Extraction and Purification

A commercial DNA extraction kit, the DNeasy® Plant Mini kit (Qiagen, Shanghai, China), was used to extract genomic DNA. 20 mg lyophilized tissue of different plant materials including fresh tobacco leaves, unprocessed tobacco, reconstituted tobacco, tobacco stems, cigarettes, and cigarette white wrappers was considered for DNA isolation. Briefly, the sample was collected and washed with 1x PBS buffer before starting DNA extraction. Then, the sample was ground in the presence of liquid nitrogen. DNA was extracted following the instructions given by the DNeasy Plant Mini kit. The qualities and quantities of extracted genomic DNA samples were measured and evaluated using the NanoDrop 1000 UV/vis Spectrophotometer (NanoDrop Technologies Inc., Wilmington, DE, USA) by observing OD260/OD280 and OD260/OD230 and 1% (w/v) agarose gel electrophoresis in 0.5x TBE with GelRed staining. All purified DNA samples were stored at −20°C until PCR analysis.

### 2.3. DNA Oligonucleotide Primers and Probes

All primers for the LAMP assay were designed with the website-based software Primer Explorer V4 (http://primerexplorer.jp/elamp4.0.0/index.html) and synthesized by Invitrogen Co., Ltd. (Shanghai, China). There are two outer and two inner primers in the LAMP assay. The two outer primers include one forward outer primer (F3) and one backward outer primer (B3) while the two inner primers include one forward inner primer (FIP) and one backward inner primer (BIP). The FIP consists of one F1c (complementary of F1) and one sense sequence F2, and the BIP consists of one B1c (complementary of B1) and one sense sequence B2 [14]. Meanwhile, DNA oligonucleotide primers and TaqMan® probes for qPCR were designed with Primer Premier 5.0 and synthesized by Invitrogen Co., Ltd. (Shanghai, China). The detailed primer and probe sequences used in this study are listed in Table S1 in Supplementary Material available online at http://dx.doi.org/10.1155/2016/4352308.

### 2.4. LAMP Assay

For the LAMP assay, each reaction was performed in a 25 *µ*L reaction mixture containing 20 mM Tris-HCl (pH 8.8), 10 mM KCl, 10 mM (NH_4_)_2_SO_4_, 25 mM MgSO_4_, 0.1% Triton X-100, 5 M betaine (Sigma, USA), 10 *µ*M of each FIP and BIP primer, 10 *µ*M of each F3 and B3 primer, 2 mM of each dNTP, and 2 *µ*L of purified genomic DNA template. After the addition of DNA template, the reaction mixture was incubated at 95°C for 5 min, followed by immediate cooling down on ice and the addition of 8 U* Bst* DNA polymerase large fragment (New England Biolabs). The reaction mixture was then incubated at 64°C for 60 min in a TaKaRa PCR Thermal Cycler (TaKaRa Bio Inc., Japan). The no template control (NTC) contained water instead of the DNA template and the LAMP assay was repeated three times. The amplified LAMP products were examined either through visual inspection with 1000x SYBR Green I (Generay Biotech Co., Ltd., Shanghai, China) or on agarose gel electrophoresis (AGE) analysis.

### 2.5. qPCR Assay

qPCR assays were performed on an ABI 7900HT Fast Real-Time PCR System (Applied Biosystems, USA) in 96-well plates. A real-time PCR reaction mixture (25 *µ*L) contained the following reagents: 12.5 *µ*L of TaqMan Gene Expression Master Mix (Applied Biosystems), 1.0 *µ*L (0.4 *µ*M) of each primer, 0.5 *µ*L (0.2 *µ*M) of the probe, 5.0 *µ*L of the ddH_2_O, and 5.0 *µ*L of the genomic DNA. Real-time PCR amplification procedures were carried out using the following conditions: one cycle of 10 min at 95°C, followed by 45 cycles of 15 s at 95°C and 60 s at 60°C. At the annealing step, the fluorescent signal was detected during every PCR cycle. PCR data was analyzed using ABI 7900HT software SDS version 2.4. The real-time PCR reaction was repeated in triplicate with every three replicates.

## 3. Results and Discussion

### 3.1. Genomic DNA Extraction

Genomic DNA from fresh leaves of different nontobacco and tobacco samples could be easily and rapidly extracted using DNeasy Plant Mini kit (Qiagen, Shanghai, China), according to the instructions given by the manufacturer, and the obtained DNA was successfully used directly for LAMP and qPCR assays, in agreement with the previous study [7]. However, the genomic DNA isolated from dried/cured tobacco leaves, reconstituted tobacco samples, tobacco stems, and cigarettes using the same kit was not pure enough for LAMP and qPCR assays. Therefore, an additional step of 2-3 times washing of those samples with 1x PBS buffer before DNA extraction was performed. We assumed that washing with PBS buffer would remove contaminants, particularly dust, inhibitory/interfering chemicals, and microorganisms that break down DNA or interfere with subsequent DNA isolation and purity. Indeed, DNA samples isolated following this strategy were found to have reliable and reproducible result in PCR amplification, both in LAMP and in real-time PCR assays.

### 3.2. Specificity Assay

The tobacco gene* UMPS* is a bifunctional enzyme that catalyzes the last two steps of the conserved pyrimidine biosynthetic pathway in higher eukaryotes including tobacco. In solanaceous species, it appears in the genome as only one copy [15]. Therefore, it was chosen in our study to investigate its suitability as a marker gene for the detection of tobacco. BLAST (Basic Local Alignment Search Tool) was then performed and a unique region for tobacco was used for the design of the suitable primer sets for both LAMP and qPCR assays (Table S1).

**Figure 1:**
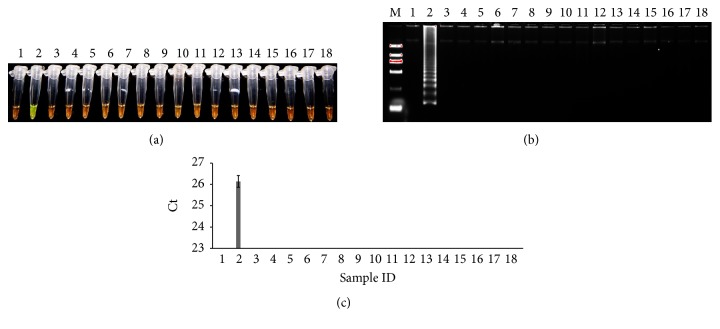
Specificity test of* UMPS* gene in tobacco and nontobacco plants. (a) LAMP method through direct visual detection with SYBR Green I; (b) LAMP method on 2% agarose gel electrophoresis analysis; (c) qPCR method.* Lane 1*: negative control (NTC);* lane 2*: positive control (PTC);* lanes 3–18*: Chinese jasmine, alfalfa,* Altingia*, canola,* Pittosporum*,* Daphniphyllum*, mondo grass, sapodilla, garden petunia, castor oil, indica rice, coriander, spinach, pomegranate, watermelon, and eggplant;* lane M*: Trans 2K DNA marker. Ct was expressed as mean Ct ± SD from 3 independent experiments with three replications.

The specificity assay of* UMPS* in LAMP and qPCR was performed in one set of plant samples containing one tobacco positive control (Table 1 and Table S2). As shown in Figure 1, LAMP detected the typical ladder-like pattern product only in the positive control, which was confirmed by the visualization test where the final green color also appeared in the positive control (Figures 1(a) and 1(b)). qPCR assay, which showed the amplification of* UMPS* only in the positive control (Figure 1(c)), also verified the specificity of* UMPS*. Therefore, both methods that were developed on* UMPS* gene were highly specific to tobacco. In this test, we also included eggplant and petunia, two plants within the same Solananceae family as tobacco. It is reported that eggplant produces nicotine as well [16]. The failure of the detection of* UMPS* in eggplant and petunia further indicated that both assays are specific to tobacco, which was also in agreement with our BLAST result.

### 3.3. Sensitivity Assay

The limit of detection (LOD) is the lowest amount or concentration of analyte that can be reliably detected through acceptable criterion [17]. To determine the LODs of the developed LAMP and qPCR assays, genomic DNA isolated from fresh tobacco leaves, unprocessed tobacco, and reconstituted tobacco samples was serially diluted to final concentrations of 60, 30, 15, 7.5, 3.75, 1.88, 0.94, and 0.47 ng/*µ*L for LAMP and 24, 12, 6, 3, 1.5, 0.75, 0.38, 0.19, 0.095, 0.048, 0.024, and 0.012 ng/*µ*L for qPCR, by 1x TE buffer, respectively. 2 *µ*L and 5 *µ*L of each diluted DNA were used as template in the LAMP assay and the qPCR assay, respectively.

**Figure 2:**
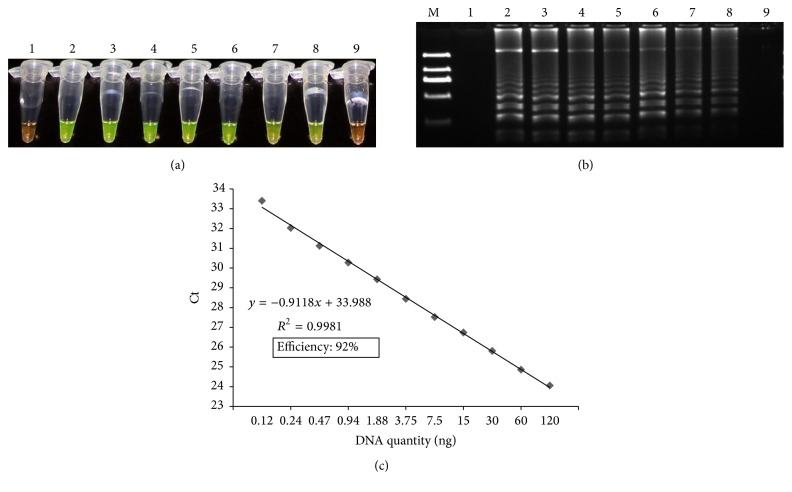
Sensitivity test of* UMPS* gene using serial dilutions of genomic DNA from fresh tobaccoleave samples. (a) LAMP method through direct visual detection with SYBR Green I; (b) LAMP method on 2% agarose gel electrophoresis analysis.* Lane 1*: NTC;* lanes 2–9*: 120, 60, 30, 15, 7.5, 3.75, 1.88, and 0.94 ng per reaction, respectively;* lane M*: Trans 2K DNA marker. (c) qPCR method. For standard curve, a serial dilution of DNA samples (120, 60, 30, 15, 7.5, 3.75, 1.88, 0.94, 0.47, 0.24, 0.12, and 0.06 ng) was used. The result was developed after considering 3 independent experiments with three replications.

As shown in Figure 2, when fresh tobacco DNA was used for the LAMP assay, the color change from orange to green was observed in all reactions of each diluted DNA level with the exception of 0.94 ng/*µ*L (Figure 2(a)). This was confirmed by the agarose gel electrophoresis analysis (Figure 2(b)), indicating that the absolute LOD of LAMP detection of* UMPS* gene was 1.88 ng (0.94 ng/*µ*L × 2 *µ*L in a reaction), which was equivalent to about an average of 395 copies of tobacco genomic DNA (http://cels.uri.edu/gsc/cndna.html). The identical LAMP results of the sensitivity assay were also obtained in assays with reconstituted tobacco samples (Figures S1A and 1B) and cured tobacco samples (Figures S2A and 2B). In the qPCR assay, standard curves were generated by plotting the average Ct values versus known quantities of genomic tobacco DNA (Figure 2(c)), and the LOD of the qPCR for* UMPS* was 0.12 ng (0.024 ng/*µ*L × 5 *µ*L in a reaction) when fresh tobacco samples were used, which was equivalent to an average of 25 copies of tobacco genomic DNA and better than the LOD reported for* PMT1a* (0.37 ng, equivalent to an average of 39 copies of tobacco genomic DNA) [7]. The LODs of the qPCR for* UMPS* in reconstituted tobacco samples and cured tobacco samples were 0.47 ng and 0.24 ng, respectively, equivalent to about an average of 100 copies and 50 copies of tobacco genomic DNA, respectively (Figures S1C and S2C). We repeated these experiments several times with serially diluted freshly isolated DNA and reproduced the results.

Our abovementioned results indicated that although the extent of processing did not affect the sensitivity of LAMP method, it did significantly affect the sensitivity of the qPCR method. It is reported that the DNA yield of processed tobacco samples differed significantly from unprocessed samples [7]. Our study, on the other hand, indicated that even if the same amount of DNA was used for the PCR reaction, the LOD of qPCR with DNA from processed tobacco samples was higher than that with DNA from unprocessed tobacco samples, demonstrating that processing affected the integrity of the genomic DNA and caused the breakdown or damage to targeted genes. In addition, our results demonstrated that the qPCR method had about 4 to 10 times lower LOD than the LAMP method.

### 3.4. Application in Various Practical Samples

**Figure 3:**
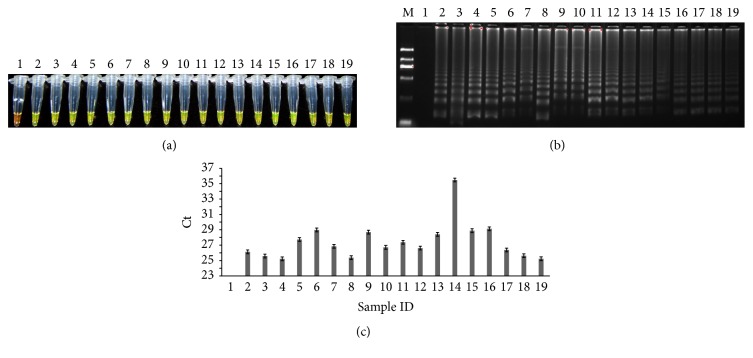
Detection of tobacco components in different tobaccocultivars. (a) LAMP method through direct visual detection with SYBR Green I; (b) LAMP method on 2% agarose gel electrophoresis analysis; (c) qPCR method.* Lane 1*: NTC;* lane 2*: PTC;* lanes 3–17*: 15 tobacco samples of different cured tobacco varieties;* lanes 18-19*: two fresh tobacco samples;* lane M*: Trans 2K DNA marker. Ct was expressed as mean Ct ± SD from 3 independent experiments with three replications.

The developed LAMP and real-time PCR methods were first used to detect the presence of the targeted gene in different cultivars of tobacco (listed in Table S2). As shown in Figure 3, it seemed that the presence of* UMPS* was conserved in these tested tobacco cultivars, and there is no single nucleotide polymorphism in this region of the gene that may negatively affect its detection among these tobacco cultivars. Thus, the result strongly suggested that* UMPS* can serve as a tobacco specific gene for the detection of tobacco components based on either LAMP or qPCR assay.

**Figure 4:**
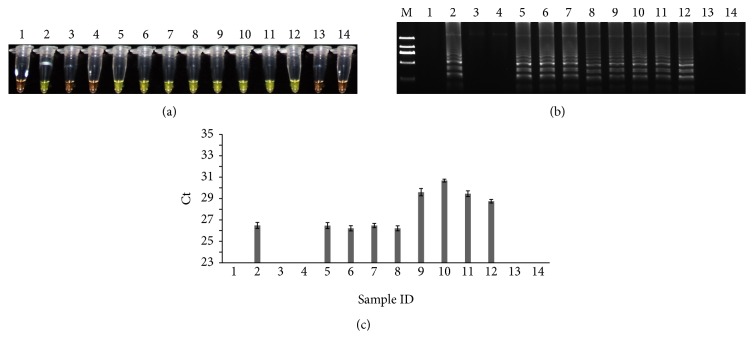
Practical sample detection with different practical samples. (a) LAMP method through direct visual detection with SYBR Green I; (b) LAMP method on 2% agarose gel electrophoresis analysis; (c) qPCR method.* Lane 1*: NTC;* lane 2*: PTC;* lanes 3-4*: coriander and spinach;* lanes 5-6*: burley and Oriental SAADI-6;* lanes 7-8*:* NT*. Oriental and* NT*. Virginia gold;* lanes 9-10*: reconstituted tobacco (no tobacco stems, no sulphate cellulose) and reconstituted tobacco (tobacco stems, with sulphate cellulose);* lanes 11-12*: cigarette (Liqun and Double Happiness);* lanes 13-14*: wrappers from Liqun and Marlboro, respectively;* lane M*: Trans 2K DNA marker. Ct was expressed as mean Ct ± SD from 3 independent experiments with three replications.

The developed LAMP and real-time PCR methods were then used to detect the presence of tobacco in various practical samples, like a positive tobacco control, a negative control, and other samples such as nontobacco samples, reconstituted tobacco samples, tobacco stems, cigarettes, and cigarette white wrappers (Table 1 and Table S3). As shown in Figure 4 and Figures S3–S5, both LAMP and qPCR methods could accurately detect the presence of tobacco in samples containing tobacco components, such as positive control, tobacco stem, reconstituted tobacco, and cigarette samples except cigarette white wrappers. These results showed that both methods were effective and reliable for the detection of the presence of tobacco components, regardless of the degree of processing, indicating that both are of high potential to be used for reliable and accurate detection of tobacco in customs service for tariff clarification and excise.

## 4. Conclusions

In this study, we have established two DNA based methods, LAMP and qPCR, to detect the presence of tobacco components. The former can be used for rapid and visual identification of tobacco components as LAMP method usually does in other DNA based detection cases [18], which is particularly useful for on-site assays, while the later can be used for accurate qualitative identification of tobacco and its products for routine analysis in the lab, which has lower LOD than the previous TaqMan method [7]. Both are applicable not only to fresh samples but also to processed samples in which tobacco morphology or chemical composition has been altered in any cultivars of tobacco. Furthermore, both tobacco specific DNA based methods differ from those smell based methods currently used in customs and could be used not only for smuggling control but also for tariff classification and for excise in tobacco trade. In the future, more tobacco and nontobacco plants and more practical samples will be tested to further confirm the specificity and applicability of both methods.

## Supplementary Material

Supplementary materials include two parts: Supplementary Figures 1-7 and Supplementary Tables 1-2. Figure S1 and S2 show the sensitivity test of UMPS gene using serial dilutions of genomic DNA extracted from reconstituted and unprocessed tobacco samples, respectively. Figure S3 exhibits the quality evaluation of extracted genomic DNA on 1% (w/v) agarose gel electrophoresis in 0.5xTBE with GelRed staining. Figure S4 shows amplification plots of qPCR used DNA from different tobacco samples. Figures S5-S7 show the detection result of tobacco components in practical samples, reconstituted tobacco samples and cigarette white wrapper, respectively. Table S1 list the primers and probes used in this study. Table S2 describes the different tobacco samples received from Bulgarian Customs.

## Figures and Tables

**Table 1:** Descriptions of plant samples used in this study.

Plant materials	Description of the plant materials	Providers
Nontobacco plants (fresh leaves)	Alfalfa (*Medicago sativa*); *Altingia* (*Altingia gracilipes*); canola (*Brassica campestris*); castor (*Ricinus communis*); Chinese jasmine (*Jasminum officinale*); coriander (*Coriandrum sativum*); *Daphniphyllum* (*Daphniphyllum teijsmannii*); eggplant (*Solanum melongena*); garden petunia (*Petunia × hybrid*); Indica rice (*Oryza sativa *var*. indica*); mondo grass (*Ophiopogon japonicus*); *Pittosporum* (*Pittosporum tobira*); pomegranate (*Punica granatum*); sapodilla (*Manilkara zapota*); spinach (*Spinacia oleracea*); watermelon (*Citrullus lanatus*)	Locally collected

Dried tobacco leaves	Burley (3); Oriental SAADI-6; Virginia (2); Oriental (5); reconstituted tobacco foiled (1); Virginia grade A (1); Virginia grade B (1); Oriental BASMAK	Bulgarian customs

Fresh tobacco leaves	Burley; Maryland	Locally collected

Reconstituted tobacco	Factory sample I (tobacco stems, no sulphate cellulose); factory sample II (no tobacco stems, no sulphate cellulose); factory sample III (tobacco stems, with sulphate cellulose); commercial wrapper	Dutch customs

Tobacco stem	Tobacco stems	Bulgarian customs

Cigarettes	Liqun, Double Happiness, Mevius, Marlboro, Wuyeshen	Locally purchased

The numbers in brackets indicate the numbers of tobacco varieties of this species tested in the experiment.
