# Supplementary material for: The Development of DNA Based Methods for the Reliable and Efficient Identification of Nicotiana tabacum in Tobacco and Its Derived Products
Source: Int J Anal Chem. 2016 Aug 18;2016:4352308. doi: 10.1155/2016/4352308 (PMC5007345; doi:10.1155/2016/4352308)
Supplement: Supplementary file 1 — Supplementary materials include two parts: Supplementary Figures 1-7 and Supplementary Tables 1-2. Figure S1 and S2 show the sensitivity test of UMPS gene using serial dilutions of genomic DNA extracted from reconstituted and unprocessed tobacco samples, respectively. Figure S3 exhibits the quality evaluation of extracted genomic DNA on 1% (w/v) agarose gel electrophoresis in 0.5xTBE with GelRed staining. Figure S4 shows amplification plots of qPCR used DNA from different tobacco samples. Figures S5-S7 show the detection result of tobacco components in practical samples, reconstituted tobacco samples and cigarette white wrapper, respectively. Table S1 list the primers and probes used in this study. Table S2 describes the different tobacco samples received from Bulgarian Customs. [file 4352308.f1.pdf]

Supplementary materials

Supplementary figures

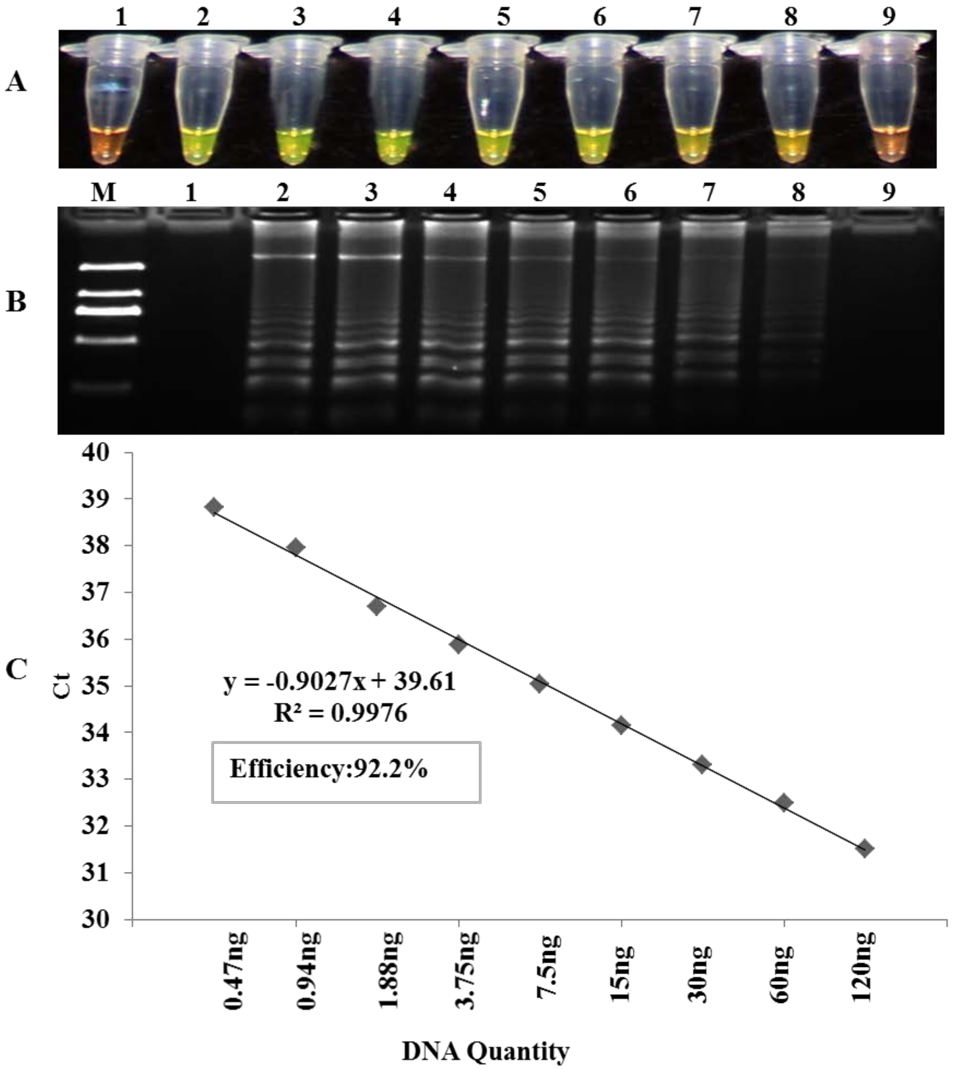

FIGURE S1: Sensitivity test of UMPS gene using serial dilutions of genomic DNA from reconstituted tobacco samples. (A) LAMP method through direct visual detection with SYBR Green I; (B) LAMP method on 2% agarose gel electrophoresis analysis. *Lane 1*, NTC; *lanes 2-9*, 120, 60, 30, 15, 7.5, 3.75, 1.88 and 0.94 ng per reaction, respectively; *lane M*, Trans 2K DNA marker. (C) qPCR method. For standard curve, a serial dilution of DNA samples (120, 60, 30, 15, 7.5, 3.75, 1.88, 0.94, 0.47, 0.24, 0.12 and 0.06 ng per reaction) was used. The result was developed after considering 3 independent experiments with three replications.

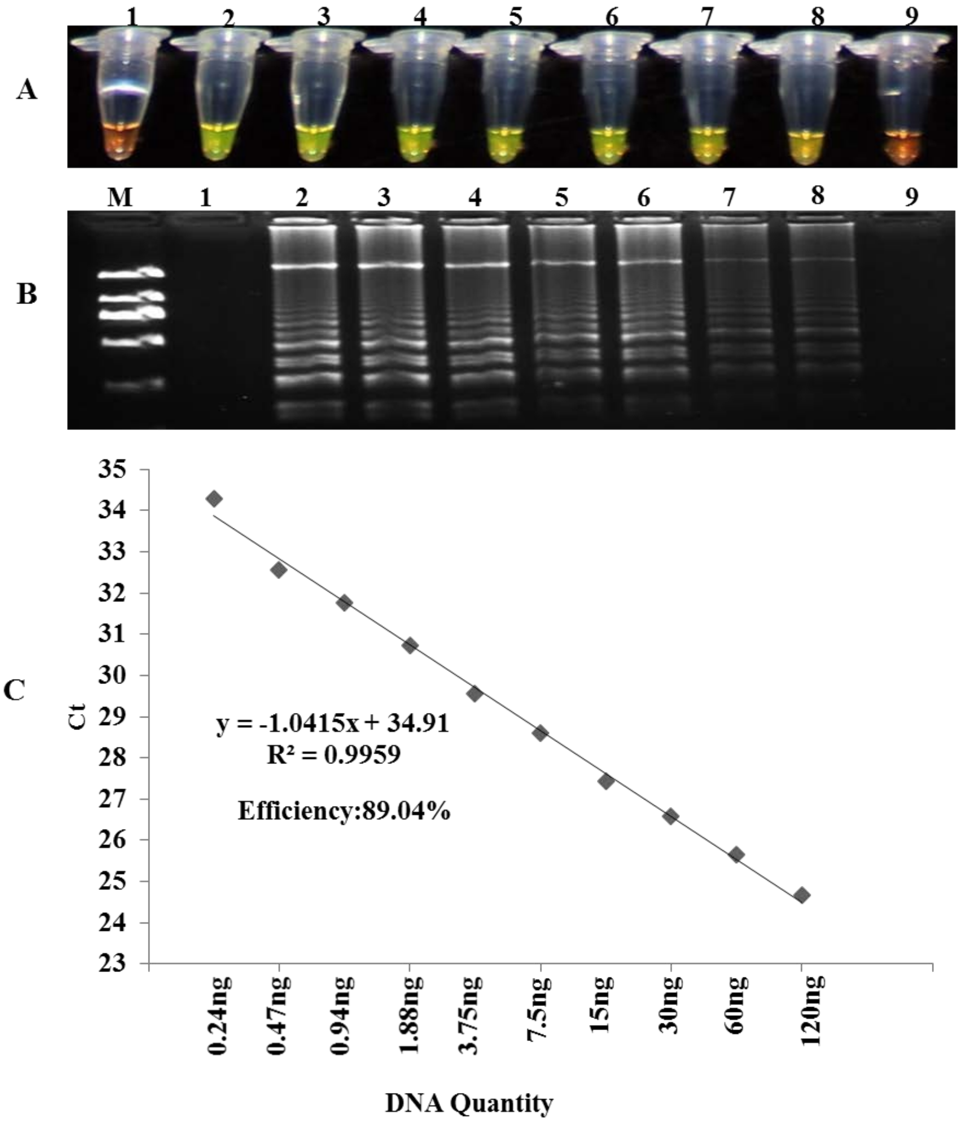

FIGURE S2: Sensitivity test of UMPS gene using serial dilutions of genomic DNA of unprocessed tobacco samples. (A) LAMP method through direct visual detection with SYBR Green I; (B) LAMP method on 2% agarose gel electrophoresis analysis. *Lane 1*, NTC; *lanes 2-9*, 120, 60, 30, 15, 7.5, 3.75, 1.88 and 0.94 ng per reaction, respectively; *lane M*, Trans 2K DNA marker. (C) qPCR method. For standard curve, a serial dilution of DNA samples (120, 60, 30, 15, 7.5, 3.75, 1.88, 0.94, 0.47, 0.24, 0.12 and 0.06 ng per reaction) was used. The result was developed after considering 3 independent experiments with three replications.

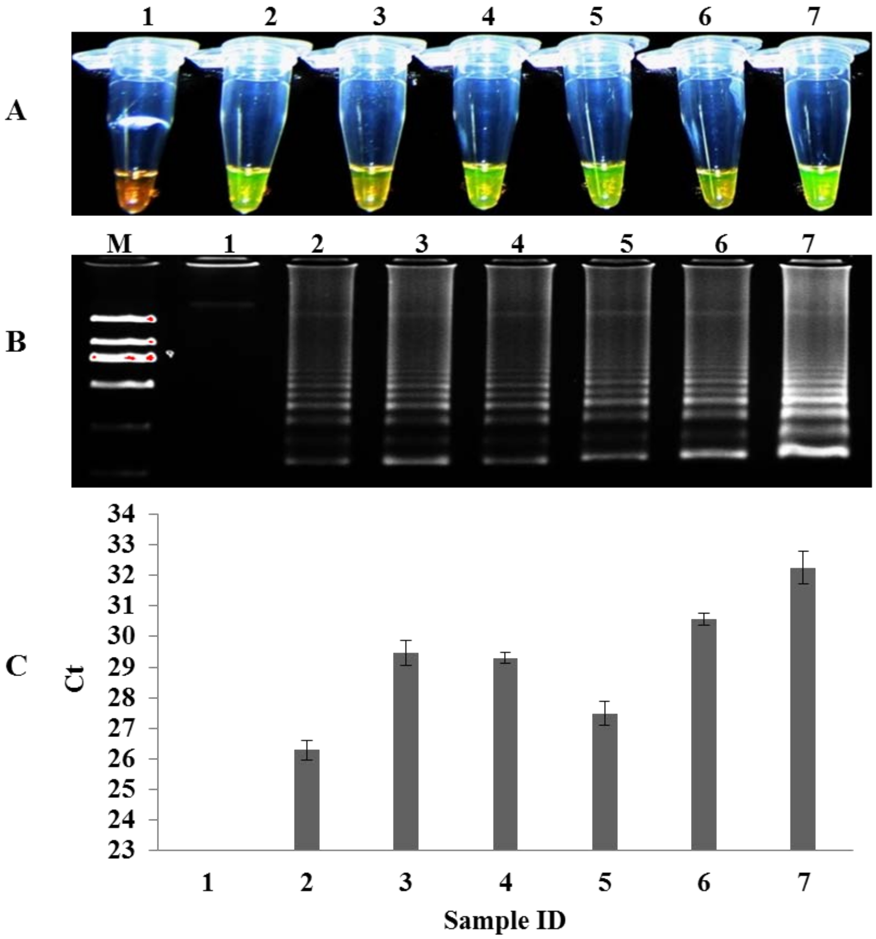

FIGURE S3: Detection of tobacco components in practical samples. (A) LAMP method through direct visual detection with SYBR Green I; (B) LAMP method on 2% agarose gel electrophoresis analysis; (C) qPCR method. *Lane 1*, NTC; *lane 2*, PTC; *lanes 3-7*, different cigarette brands like liqun, double happiness, mevius, marlboro and wuyeshen; *lane M*, Trans 2K DNA marker. Ct was expressed as mean Ct ±SD from 3 independent experiments with three replications.

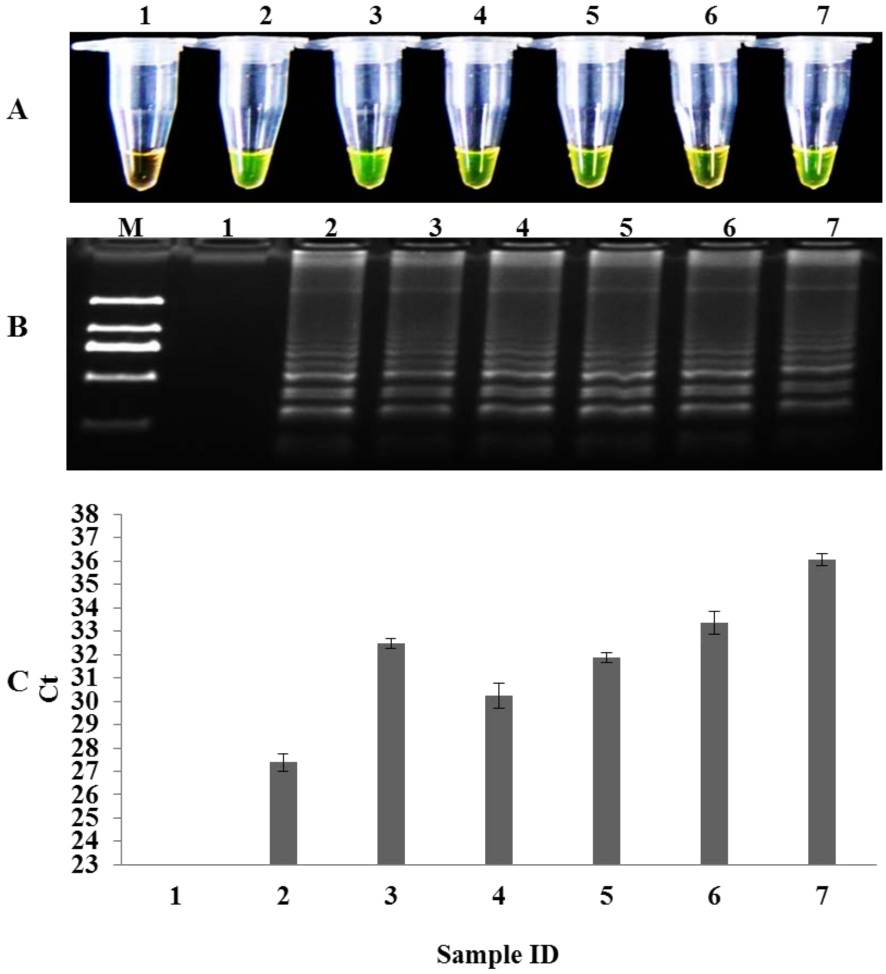

FIGURE S4: Detection of tobacco components in reconstituted tobacco samples. (A) LAMP method through direct visual detection with SYBR Green I; (B) LAMP method on 2% agarose gel electrophoresis analysis; (C) qPCR detection. *Lane 1*, NTC; *lane 2*PTC; *lanes 2-6,* different reconstituted tobacco samples, *lane 7*, tobacco stems sample; *lane M*, Trans 2K DNA marker. Ct was expressed as mean Ct ±SD from 3 independent experiments with three replications.

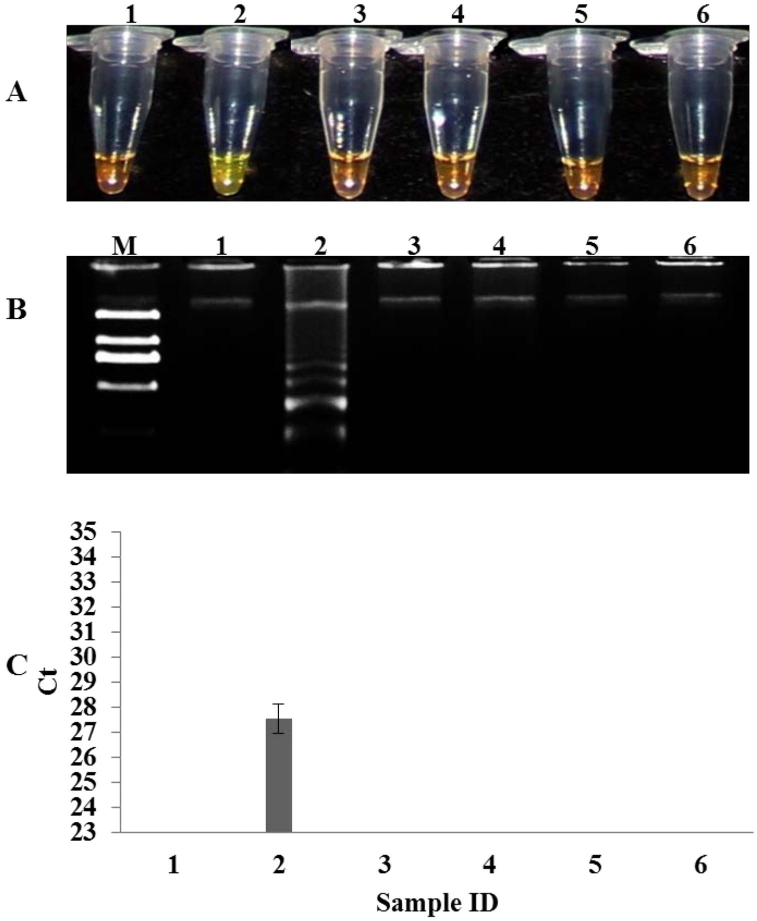

FIGURE S5: Detection of tobacco components in cigarette white wrapper. (A) LAMP method through direct visual detection with SYBR Green I; (B) LAMP method on 2% agarose gel electrophoresis analysis; (C) qPCR detection. *Lane 1*, NTC; *lane 2*, PTC; *lanes 3-6*, Cigarettes wrappers from Liqun, Double happiness, Mevius and Marlboro, respectively; *lane M*, Trans 2K DNA marker. Ct was expressed as mean Ct ±SD from 3 independent experiments with three replications.

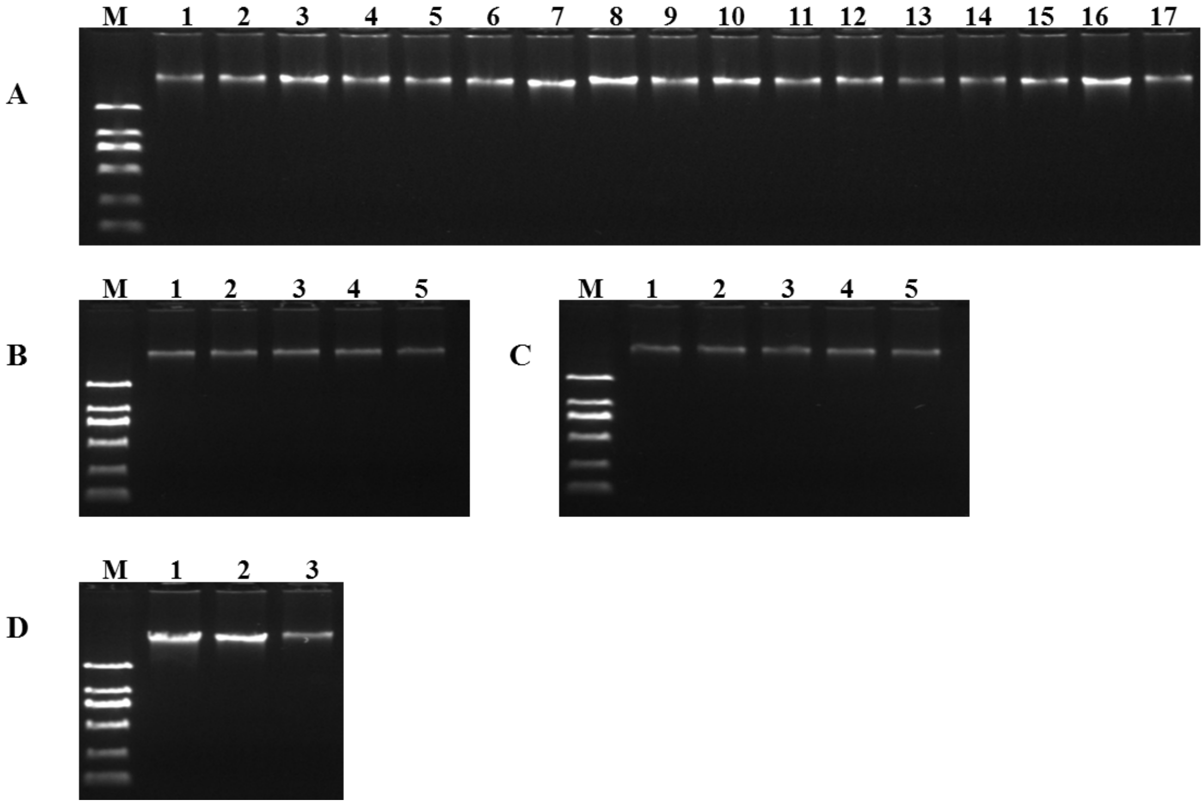

FIGURE S6: Qualities evaluation of extracted genomic DNA on 1% (w/v) agarose gel electrophoresis in 0.5×TBE with GelRed staining. (A) Different tobacco samples received from Bulgarian customs. (B) Different cigarette samples purchased from locally. (C) Different reconstituted tobacco samples received from Netherlands and Bulgarian customs. (D) Different tobacco samples (fresh leaves, dried leaves and reconstituted tobacco) used for determination of the LOD.

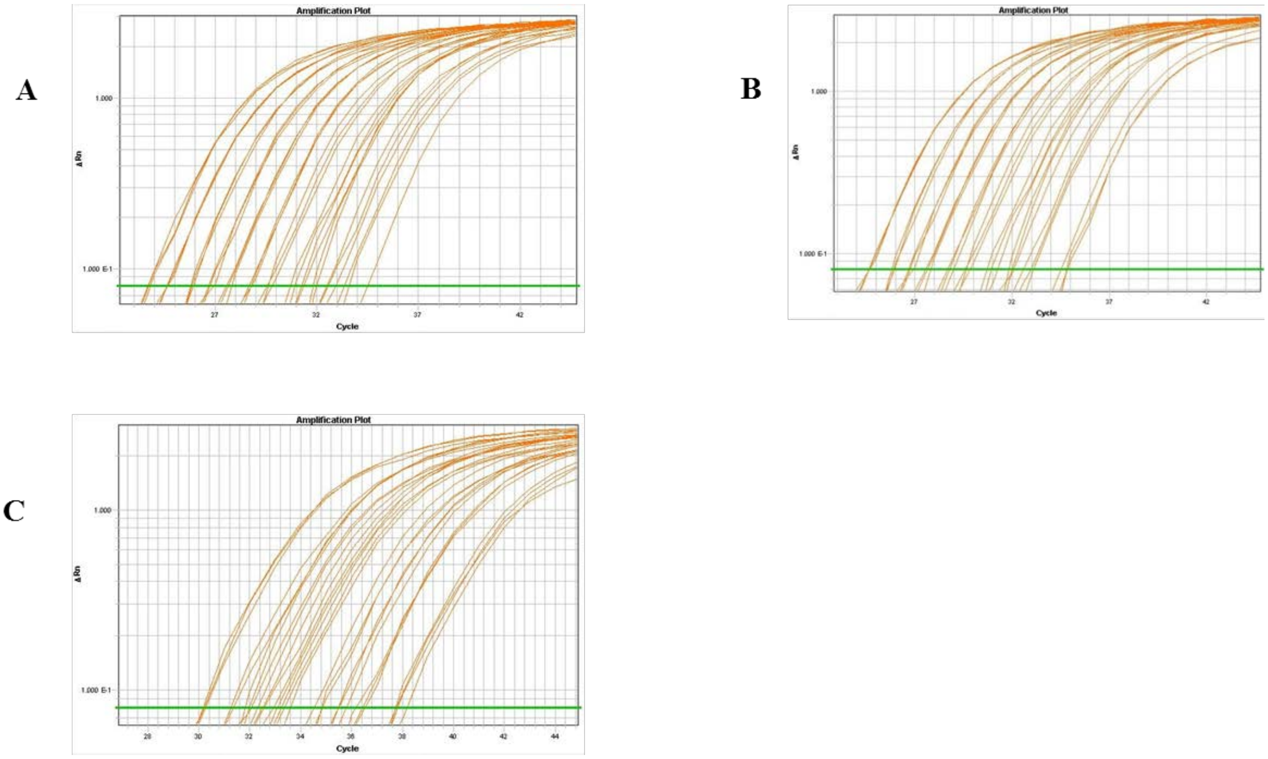

FIGURE S7: Amplification plots of qPCR used DNA from different tobacco samples. (A) Fresh tobacco leaves. (B) Unprocessed tobacco leaves. (C) Reconstituted tobacco samples.

Supplementary tables

TABLE S1: List of oligonucleotide primers and probes used in this study

PCR type	Primer/probe name	Sequence (5'----3')	Amplicon size
Real-time PCR	Q-UMPS-F	TGCAAGAAGAATGTGATAGTAAGTG	82 bp
	Q-UMPS-R	ACTCCAACTTCTACAAGCTATCC	
	Q-UMPS-P	CAGCTGTGTAGGGTATAGTGCATTT	
LAMP	UMPS-F3	TCACAAGGTTTGTAGAGACTT	222 bp
	UMPS-B3	CACACAAACATAAATCATTGGAC	
	UMPS-FIP	ACTTTGATACCTTTGTAAGCCCCACCTTGTCTTTGGGTGTCG	
	UMPS-BIP	ATGAATTGTCCTAAACCTCCTTTGTAAGTAGGAAATGTCGCCTG	

TABLE S2: Description of different tobacco samples received from Bulgarian Customs.

Sample ID	Type/sort of tobacco acc. documents by import	Country of origin	Type of Curing
01_2014	Burley	India	light air-cured
02_2014	Oriental SAADI-6	Lebanon	sun cured Oriental type
03_2014	Virginia	India	flue-cured
04_2014	Burley	India	light air-cured
05_2014	Virginia	India	flue-cured
06_2014	Burley	Serbia	light air-cured
07_2014	Oriental	Bulgaria	sun cured Oriental type
08_2014	Oriental	Bulgaria	sun cured Oriental type
09_2014	Oriental	Bulgaria	sun cured Oriental type
10_2014	Oriental	Bulgaria	sun cured Oriental type
11_2014	Oriental	Bulgaria	sun cured Oriental type
12_2014	Reconstituted tobacco foil	unknown	n.a.
13_2014	Virginia grade A	India	flue-cured
14_2014	Virginia grade B	India	flue-cured
15_2014	Oriental BASMAK	India	sun cured Oriental type

TABLE S3: Description of reconstituted tobacco samples received from Netherlands and Bulgarian Customs.

Sample ID	Descriptions of reconstituted tobacco
Sample 1	Reconstituted tobacco, factory sample: tobacco stems, no sulphate cellulose
Sample 2	Reconstituted tobacco, factory sample: no tobacco stems, no sulphate cellulose
Sample 3	Reconstituted tobacco, factory sample: tobacco stems, with sulphate cellulose
Sample 4	Reconstituted tobacco: commercial wrapper
Sample 5	Tobacco Stems (Bulgarian Customs)
